# Gun-carrying restrictions and gun-related mortality, Colombia: a difference-in-difference design with fixed effects

**DOI:** 10.2471/BLT.19.236646

**Published:** 2019-01-17

**Authors:** Andres I Vecino-Ortiz, Deivis N Guzman-Tordecilla

**Affiliations:** a Department of International Health, Johns Hopkins School of Public Health, 615 N Wolfe Street, Suite E8620, Baltimore, Maryland 21205, United States of America.; b Red Papaz, Bogotá, Colombia.

## Abstract

**Objective:**

To assess the effect of a permanent gun-carrying restriction on gun-related mortality in Colombia between 2008 and 2014, and determine differences in the effect of the restriction by place of death and sex.

**Methods:**

In 2012, Bogotá and Medellín introduced a permanent gun-carrying restriction. We compared gun-related mortality rates in these cities (intervention cities) with the rates in all other Colombian cities with more than 500 000 inhabitants (control cities). We used data from the Colombian National Department of Statistics to calculate monthly gun-related mortality rates between 2008 and 2014 for intervention and control cities. We used a differences-in-differences method with fixed effects to assess differences in gun-related mortality in intervention and control cities before and after the introduction of the gun-carrying restriction. We stratified effects by place of death (public area or residence) and sex. We made robustness checks to test the assumptions of the models.

**Findings:**

Gun-related deaths in the control and intervention cities decreased between 2008 and 2014; however, the decrease was greater in the intervention cities (from 20.29 to 14.93 per 100 000 population; 26.4%) than in the control cities (from 37.88 to 34.56 per 100 000 population; 8.8%). The restriction led to a 22.3% reduction in the monthly gun-related mortality rate in Bogotá and Medellín. The reduction was greater in public areas and for males. Robustness checks supported the assumptions of the models.

**Conclusion:**

The permanent restriction on carrying guns reduced gun-related deaths. This policy could be used to reduce gun-related injuries in urban centres of other countries with large numbers of gun-related deaths.

## Résumé

**Objectif:**

Évaluer l'impact d'une restriction permanente du port d'armes sur le taux de mortalité lié aux armes à feu en Colombie entre 2008 et 2014, et déterminer les différences d'impact de cette restriction en fonction du lieu du décès et du sexe.

**Méthodes:**

En 2012, Bogotá et Medellín ont instauré une restriction permanente du port d'armes. Nous avons comparé les taux de mortalité liés aux armes à feu dans ces villes (villes d'intervention) avec les taux observés dans l'ensemble des autres villes colombiennes de plus de 500 000 habitants (villes de contrôle). Les données fournies par le Département administratif national de la statistique en Colombie nous ont permis de calculer les taux de mortalité liés aux armes à feu entre 2008 et 2014 dans les villes d'intervention et de contrôle. Nous avons utilisé la méthode des doubles différences à effets fixes pour mesurer les disparités entre la mortalité liée aux armes à feu dans les villes d'intervention et de contrôle, avant et après l'instauration de la restriction du port d'armes. Nous avons ensuite segmenté les résultats en fonction du lieu du décès (espace public ou domicile) et du sexe. Nous avons également procédé à des tests de robustesse pour évaluer les hypothèses des modèles.

**Résultats:**

Les décès causés par des armes à feu dans les villes de contrôle et d'intervention ont diminué entre 2008 et 2014. Cependant, la diminution était plus importante dans les villes d'intervention (de 20,29 à 14,93 par 100 000 habitants ; 26,4 %) que dans les villes de contrôle (de 37,88 à 34,56 par 100 000 habitants ; 8,8 %). La restriction a entraîné une baisse de 22,3 % de la mortalité mensuelle liée aux armes à feu, tant à Bogotá qu'à Medellín. Cette baisse s'est avérée plus importante dans les espaces publics et pour les individus de sexe masculin. Les tests de robustesse ont confirmé les hypothèses des modèles.

**Conclusion:**

La restriction permanente du port d'armes a un impact positif sur la mortalité liée aux armes à feu. Cette politique pourrait être employée par d'autres pays où le nombre de décès attribuables aux armes à feu est élevé, afin de réduire le nombre de blessures par balles dans les centres urbains.****

## Resumen

**Objetivo:**

Evaluar el efecto de una restricción permanente al porte de armas en la mortalidad relacionada con las armas en Colombia entre 2008 y 2014, y determinar las diferencias en el efecto de la restricción por lugar de muerte y sexo.

**Métodos:**

En 2012, Bogotá y Medellín establecieron una restricción permanente al porte de armas. Se compararon las tasas de mortalidad por arma de fuego en estas ciudades (ciudades de intervención) con las tasas en todas las demás ciudades colombianas con más de 500.000 habitantes (ciudades de control). Se utilizaron los datos del Departamento Nacional de Estadística de Colombia para calcular las tasas mensuales de mortalidad por armas de fuego entre 2008 y 2014 para las ciudades de intervención y control. Se utilizó un método de diferencia en diferencias con efectos fijos para evaluar las diferencias en la mortalidad relacionada con armas de fuego en las ciudades de intervención y control antes y después del establecimiento de la restricción al porte de armas. Se estratificaron los efectos por lugar de muerte (área pública o residencia) y sexo. Se realizaron controles de solidez para probar los supuestos de los modelos.

**Resultado:**

Las muertes relacionadas con armas de fuego en las ciudades de control e intervención disminuyeron entre 2008 y 2014; sin embargo, la disminución fue mayor en las ciudades de intervención (de 20, 29 a 14,93 por cada 100.000 habitantes; 26,4 %) que en las ciudades de control (de 37,88 a 34,56 por cada 100.000 habitantes; 8,8 %). La restricción condujo a una reducción del 22,3 % en la tasa mensual de mortalidad relacionada con armas de fuego en Bogotá y Medellín. La reducción fue mayor en las áreas públicas y en los hombres. Los controles de solidez apoyaron los supuestos de los modelos.

**Conclusión:**

La restricción permanente al porte de armas redujo las muertes relacionadas con las armas. Esta política podría ser utilizada para reducir las lesiones relacionadas con armas de fuego en los centros urbanos de otros países con un gran número de muertes relacionadas con este tipo de armas.

## ملخص

**الغرض:**

تقييم تأثير التقييد الدائم لحمل السلاح على الوفيات المرتبطة بالأسلحة في كولومبيا بين عامي 2008 و2014، وتحديد الاختلافات في تأثير التقييد حسب مكان الوفاة والجنس.

**الطريقة:**

قامت كل من بوجوتا وميديلين في عام 2012، بفرض تقييد دائم على حمل السلاح. وقمنا بمقارنة معدلات الوفيات المرتبطة بالأسلحة في هاتين المدينتين (مدن التدخل)، بالمعدلات المقابلة لها في جميع المدن الكولومبية الأخرى التي يزيد عدد سكانها عن 500000 نسمة (مدن السيطرة). وقمنا بالاستعانة بالبيانات من إدارة الإحصاءات الوطنية الكولومبية لحساب معدلات الوفيات الشهرية المرتبطة بالسلاح بين عامي 2008 و2014 في مدن التدخل والسيطرة. كما استخدمنا طريقة الاختلافات في الفارق ذات التأثيرات الثابتة، لتقييم الاختلافات في الوفيات المرتبطة بالأسلحة النارية في مدن التدخل والسيطرة قبل وبعد فرض قيود حمل السلاح. وقمنا بتصنيف التأثيرات حسب مكان الوفاة (المنطقة العامة أو مقر الإقامة) والجنس. كما أجرينا فحوصات فعالية لاختبار افتراضات النماذج.

**النتائج:**

انخفض معدل الوفيات المرتبطة بالأسلحة في مدن السيطرة والتدخل بين عامي 2008 و2014؛ إلا أن الانخفاض كان أكبر في مدن التدخل (من 20.29 إلى 14.93 لكل 100000 نسمة؛ 26.4%)، منه في مدن السيطرة (من 37.88 إلى 34.56 لكل 100000 نسمة؛ 8.8%). أدى التقييد إلى انخفاض بنسبة 22.3% في معدل الوفيات الشهرية المرتبطة بالسلاح في بوجوتا وميديلين. كان الانخفاض أكبر في المناطق العامة، وبالنسبة للذكور. كما أيدت فحوصات الفعالية افتراضات النماذج.

**الاستنتاج:**

أدى التقييد الدائم على حمل السلاح إلى خفض الوفيات المرتبطة بالأسلحة. يمكن استخدام هذه السياسة للحد من الإصابات المرتبطة بالأسلحة في المراكز الحضرية في البلدان الأخرى ذات الأعداد الكبيرة من الوفيات المرتبطة بالأسلحة. ****

## 摘要

**目的:**

旨在评估 2008 年至 2014 年哥伦比亚实施的永久性持枪限制对涉枪死亡率的影响，并根据死亡地点和性别确定限制效果的差异。

**方法:**

2012 年，波哥大和梅德林实施了永久性持枪限制。我们比较了这两座城市（干预城市）与居住人口超过 500 000 的所有其他哥伦比亚城市（控制城市）的涉枪死亡率。我们使用哥伦比亚国家统计局的数据，计算 2008 年至 2014 年干预城市和控制城市每月涉枪事件的死亡率。我们采用具有固定效应的倍差法来评估实施持枪限制前后干预城市和控制城市涉枪死亡率的差异。我们根据死亡地点（公共区域或居民区）和性别对影响进行分层。我们开展了稳健性检验来验证模型的假设。

**结果:**

2008 年至 2014 年间，控制城市和干预城市中涉枪事件的死亡人数有所下降；但干预城市的下降幅度（从每 100 000 人口 20.29 下降至 14.93；26.4%）大于控制城市（从每 100 000 人口 37.88 下降至 34.56；8.8%）。这一限制导致波哥大和梅德林每月涉枪死亡率下降 22.3%。这一数字在公共区域和男性中的减少幅度更大。稳健性检验支持模型的假设。

**结论:**

对携带枪支的永久性限制减少了涉枪死亡率。对于涉枪死亡人数较多的其他国家而言，这项政策可以用来减少其城市中心地区涉枪事件带来的损伤。****

## Резюме

**Цель:**

Оценить влияние постоянного ограничения на ношение оружия на уровень смертности от огнестрельных ранений в Колумбии в период с 2008 по 2014 годы и определить различия в эффективности ограничения в зависимости от места гибели и пола жертвы.

**Методы:**

В 2012 году в городах Богота и Медельин были введены постоянные ограничения на ношение оружия. Авторы сравнили уровень смертности от огнестрельного оружия в этих городах (города проведения вмешательства) с показателями в других колумбийских городах с населением более 500 000 жителей (контрольные города). Для расчета показателей смертности от огнестрельных ранений в городах проведения вмешательства и контрольных городах использовались данные Национального административного департамента статистики Колумбии за период с 2008 по 2014 годы. Авторы применили метод «разность разностей» с фиксированными эффектами для оценки разницы в показателях смертности от огнестрельных ранений в городах проведения вмешательства и контрольных городах до и после вступления в силу ограничений на ношение оружия. Эффекты были стратифицированы по месту гибели (в общественном месте или дома) и полу жертв. Для проверки модельных предположений авторы провели проверку надежности результатов.

**Результаты:**

Уровень смертности от огнестрельных ранений в период с 2008 по 2014 годы снизилась как в городах проведения вмешательства, так и в контрольных городах; однако в городах проведения вмешательства снижение было значительнее (с 20,29 до 14,93 человека на 100 000 населения; 26,4%), чем в контрольных городах (с 37,88 до 34,56 человека на 100 000 населения; 8,8%). Ежемесячный показатель смертности от огнестрельных ранений в городах Богота и Медельин вследствие введенного ограничения снизился на 22,3%. Снижение было значительнее выражено для общественных мест и для мужчин. Проверка надежности результатов подтвердила основные предположения модели.

**Вывод:**

Постоянное ограничение на ношение оружия уменьшило количество погибших от огнестрельных ранений. Этот подход может использоваться для уменьшения травматизма от огнестрельных ранений в городских центрах других стран с высокими показателями смертности от огнестрельных ранений.****

## Introduction

Estimations show that 251 000 people died in 2016 from gun-related injuries worldwide and nine out of 10 violent deaths take place outside of conflict situations.^1^^,^^2^ About 64% (161 000) of all gun-related deaths were homicides, 27% (67 500) were suicides and 9% (22 900) were ruled as unintentional.^2^ These figures highlight a growing public health problem that has social and economic costs that extend beyond the immediate loss of life.^2^ Research has shown that gun-related deaths cost around 100 billion United States dollars a year in the United States of America when taking account of societal costs, including legal procedures, loss to quality of life, psychological effects and reductions in property value.^4^

As with many other types of violence that lead to injury and death, gun-related deaths are a public health problem and are strongly associated with lower socioeconomic conditions.^5^ Among other factors, evidence from 130 studies in 10 countries suggests that access to firearms has an important effect on gun-related injuries at the population level.^6^ Some studies have identified a positive relationship between access to firearms and gun-related injuries, particularly in high-income countries.^6^^–^^11^

Gun-related injuries are an increasingly important problem in Latin America. About 35.8% (89 790/251 000) of all firearm-related deaths in the world took place in only five Latin American countries (in order of the number of deaths): Brazil, Mexico, Colombia, Bolivarian Republic of Venezuela and Guatemala.^2^ Colombia has the fifth highest number of gun-related deaths in the world after Brazil, United States of America, India and Mexico.^2^ Gun-related injuries are a leading cause of death and injury in Colombia, accounting for 9.3% (520 706/5 587 652) of all years of life lost between 1990 and 2017.^12^

In November 2016, the Colombian government and the Revolutionary Armed Forces of Colombia (FARC), a guerrilla group, signed a peace agreement, which led to a reduction in the overall number of gun-related deaths associated with the conflict.^13^ However, in urban areas, gun-related violence remains high and needs to be addressed.^12^^,^^13^ A policy option to reduce gun-related deaths in urban settings is reducing access to guns through gun-control laws, particularly through restrictions on carrying guns.^7^^,^^11^^,^^14^^,^^15^

Evidence of the effectiveness of restrictions on carrying guns in middle-income countries is scarce.^16^^–^^18^ However, the existing evidence on such restrictions suggests that they are associated with a reduction in gun-related deaths. In South Africa, the introduction of a firearm control act was estimated to have saved 4585 lives from 2001 to 2005.^19^ In Brazil, a national restriction on carrying guns saved 2800 lives in the state of São Paulo alone.^20^^,^^21^

In Colombia, temporary restrictions on carrying guns have been enacted in the past. Research on the effect of these restrictions on mortality in the mid-1990s showed average reductions in homicides in Bogotá and Cali of about 14% (rate ratio 0.86, 95% confidence interval: 0.76–0.97).^22^^–^^25^ Initially, restrictions were temporary, on specific dates, such as election season or in specific neighbourhoods with a high prevalence of violent crime.^26^^,^^27^ However, in 2012, two cities, Bogotá and Medellín, introduced a permanent city-wide restriction on carrying guns.

In this study, we aimed to assess: (i) the effect of the permanent restriction on carrying guns on gun-related deaths in the two cities where this measure was enacted and (ii) the difference in the effect of the restriction by place of death and sex of the victim.

## Methods

### Data

We used individual-level mortality data from the Colombian National Department of Statistics (DANE in Spanish) for 2008 to 2016.^28^ This data set contains anonymized information on all reported deaths in the country between 2008 and 2016, including month and year of death, basic cause of death, municipality of residence of the victim and place of death, sex of the deceased person and area (urban and rural). However, the data set does not contain some important variables such as education level. The basic cause of death is coded using the *International classification of diseases and related health problems*, version 10 (ICD 10).^29^ The database included 1 845 651 deaths between 2008 and 2016. For our study, gun-related mortality comprised all deaths associated with a gun, including suicides, homicides and unintentional incidents (ICD-10 codes: X93, X94, X95, Y22, Y23, Y24, W32, X72, X73, X74).

For the differences-in-differences analyses with fixed effects, we excluded data from 2015 and 2016 because the ceasefire and the peace agreement between the Colombian Government and the FARC guerrillas took place during this time^30^ and inclusion of data from these years could have led to overestimating estimators for effect size. Even though we assessed the effects of the restriction on carrying guns only in urban settings, where deaths related to the armed conflict were rare in the period we assessed, the presence of unobservable factors related to the ceasefire could possibly have affected gun-related mortality in large urban centres. Therefore, we decided to restrict our analysis to the period 2008 to 2014, so as not to affect our assumptions that there were no unobservable time-varying differences between the intervention and control cities.^31^

Next, we calculated gun-related crude monthly mortality rates for all cities in Colombia with more than 500 000 people in 2008,^32^ when the study period started. These cities included Bogotá and Medellín, which had enacted a permanent restriction on carrying guns (intervention cities), and seven other cities that had no restriction (control cities). We included only cities with more than 500 000 people to allow for valid comparisons between the intervention and control cities for gun-related mortality in large urban centres.^33^ We did not consider including deaths in smaller towns or rural areas, where gun-related deaths have traditionally been more closely related to the armed conflict with the guerrilla and paramilitary groups, because the intervention and control settings would not be comparable. The seven cities included as controls based on their population size in 2008^32^ were: Cali (population 2 194 781), Barranquilla (1 170 940), Cartagena (922 859), Cúcuta (606 021), Bucaramanga (521 435), Ibagué (515 424) and Soledad (505 612).

### Analysis

Bogotá and Medellín enacted a city-wide and permanent restriction on carrying guns on 1 February 2012 and 6 January 2012, respectively. We did a difference-in-differences regression analysis of the logarithm of the monthly mortality rate in the intervention and control cities including fixed effects for city and month (all months between January 2008 and December 2014), and calendar month dummies (to control for seasonality). We used the statistical package Stata 15 (StataCorp LLC, College Station, USA) for the analysis. For months with zero deaths, the logarithm of the monthly mortality rate was given a zero value to prevent those data points from being converted into missing values.^34^ Error terms were clustered at the city and month level.^31^^,^^35^

Furthermore, we compared the difference in gun-related deaths in residences (houses/homes) and public areas (excluding deaths in a hospital as we could not determine where the gun injury took place), and in males and females, in the intervention and control cities.

### Robustness checks

Our difference-in-differences method combined with fixed effects reduces the risk of time-invariant confounding factors. However, time-varying factors could still introduce bias to our estimations. Therefore, we did robustness checks to ensure that we had not misidentified diverging or converging long-term trends in the intervention and control cities that would violate the assumption of parallel trends of the difference-in-differences method. First, we did a subanalysis of a narrower period (2011 to 2013) in which we would expect the gun-carrying restriction to remain significant and relevant if the policy had an effect starting in 2012. This analysis reduces possible unwanted influences from the earlier and later years.

We also included city-specific time trends,^36^^,^^37^ to evaluate differences in time trends for each city. If the restriction policy is effective, these differences must remain significant (even though the regression coefficient itself cannot be interpreted easily as it represents the interaction of the intervention cities with each month observed).

Finally, we compared trends in the mortality rate in intervention and control cities between 2008 and 2012 (including the preintervention period only) and from 2012 to 2014 (including the postintervention period only). If the policy is the only reason why trends in gun-related mortality between intervention and control cities differed after enactment of the policy, then there should be no significant differences between the cities. Finding no significant difference strengthens the argument that there are no time-varying confounding factors in the mortality trends.

## Results

Between 2008 and 2016, the gun-related mortality rates decreased from 31.84 per 100 000 population to 18.43 per 100 000 population in Colombia. The decrease was two times greater in the intervention cities (from 20.29 per 100 000 population to 12.75 per 100 000 population; 37.2%) than control cities (from 37.88 per 100 000 population to 31.20 per 100 000 population; 17.6%; Table 1).

**Table 1:** Annual gun-related mortality rates nationally, and in intervention and control cities, Colombia, 2008–2016

Year	Gun-related mortality rate, per 100 000 population		
	National	Intervention cities^a^	Control cities^b^
2008	31.84	20.29	37.88
2009	36.66	33.77	43.25
2010	33.90	31.69	41.98
2011	30.09	26.72	40.48
2012	27.92	19.67	41.88
2013	25.39	16.20	41.86
2014	21.09	14.93	34.56
2015	19.54	12.11	31.10
2016	18.43	12.75	31.20

^a^ Intervention cities were Bogotá and Medellín.

^b^ Control cities were all other cities in Colombia with a population of more than 500 000 in 2008, which were, in order of population size: Cali, Barranquilla, Cartagena, Cúcuta, Bucaramanga, Ibagué and Soledad.

In the difference-in-differences analysis, average monthly gun-related mortality rates in the intervention cities decreased by 22.3% after the restriction was enacted (Table 2). The effect of the restriction on the gun-related mortality rate was greater in public areas (22.4%) than residences (18.3%), and for males than females. The restriction reduced gun-related deaths in males by 22.3% in public places, and 14.5% in residential settings. However, the restriction reduced gun-related deaths in females by only 6.3% in residential areas and not at all in public areas.

**Table 2:** Difference-in-differences analysis with fixed effects of the effect of the restriction on carrying guns on gun-related mortality for place of death and sex, Colombia, 2008–2014

Variable	Effect of policy *β* coefficient (SE)	*P*	Relative effect of policy on mean monthly gun-related mortality rate, %	*P*
**Overall**	−0.49 (0.15)	0.01	−22.33	< 0.05
**Public areas**				
Overall	−0.49 (0.15)	0.01	−22.44	< 0.01
Male	−0.49 (0.14)	< 0.01	−22.33	< 0.01
Female	−0.11 (0.16)	0.52	N/A^a^	N/A^a^
**Residences**				
Overall	−0.40 (0.07)	< 0.01	−18.33	< 0.01
Male	−0.32 (0.02)	< 0.01	−14.50	< 0.01
Female	0.14 (0.05)	0.03	6.28	< 0.02

SE: standard error, N/A: not applicable.

^a^ N/A as there was no statistically significant difference.

Notes: The outcome is the logarithm of the monthly mortality rates per 100 000 population between 2008 and 2014. Gun-related deaths include homicides, suicides and accidents. Public areas are public spaces such as the street. Residences refers to homes and more generally, residential areas. All models are a difference-in-differences panel design with city and month fixed effects for all Colombian cities with a population of more than 500 000 in 2008. All models include calendar month dummies. The *β* coefficients represent the interaction of a dummy variable accounting for the month of the enactment and a dummy variable identifying the treatment city. Relative effect represents the percentage difference between intervention and control cities, before and after the intervention when divided by the mean monthly mortality rate at the time of the enactment. Mean monthly mortality rate of the total sample at the time of the enactment of the restriction was 2.18 deaths per 100 000 population. Number. of observations was 756.

Table 3 shows the robustness checks. When we assessed the effect of the gun-carrying restriction on gun-related mortality over a shorter time period (2011–2013) and on city-specific time trends, the differences after enactment of the policy were still statistically significant (*P* < 0.01). In addition, we found no statistically significant differences in gun-related mortality between the intervention and control cities when assessing only the preintervention or the postintervention period, suggesting that trends in the intervention and control cities are parallel outside of the enactment of the gun carrying-restriction.

**Table 3:** Robustness check of the effect of the restriction on carrying guns on gun-related mortality, Colombia, 2008–2014

Time period	No. of observations	Effect of policy, *β* coefficient (SE)	*P*
2011–2013 (gun-related mortality)	323	−0.39 (0.10)	< 0.01
2008–2011 (gun-related mortality)	432	0.28 (0.58)	0.64
2012–2014 (gun-related mortality)	324	−0.13 (0.46)	0.77
2008–2014 (city-specific time trends)	756	−2.51 × 10^−5^ (7.85 × 10^−6^)	0.01

SE: standard error.

Notes: The outcome is the logarithm of the monthly mortality rate per 100 000 population associated with gun injuries. Gun-related deaths include homicides, suicides and unintentional incidents. All models are a difference-in-differences panel design with city and the month fixed effects for all Colombian cities with a population of more than 500 000 in 2008. All models include calendar month dummies. The *β* coefficients represent the interaction of a dummy variable accounting for month of the enactment and a dummy variable identifying the treatment city. The coefficients for the periods before and after enactment of the restriction compare the intervention and the control cities. The coefficient for the city-specific time trends model represents an interaction of a dummy variable accounting for the month of the enactment, a dummy variable identifying the treatment city and the time variable accounting for every month in the study period.

## Discussion

We aimed to identify the effects of a permanent restriction on carrying guns on gun-related mortality in two cities in Colombia. We found that the gun-carrying restriction reduced mortality rates in both cities by a fifth. This percentage represents a reduction of about 30 deaths a month in both intervention cities. This is also a lost opportunity of preventing the loss of about 45 lives a month in the seven control cities (taking as baseline the aggregated number of deaths in intervention and control cities in the month before enactment of the restriction).

Furthermore, the effect of the gun-carrying restriction on gun-related mortality was greater in public areas than in residences. This difference is expected because the restriction prevents gun-carrying (carrying guns in public areas) not gun ownership (owning a gun and having it at home). Nonetheless, the fact that gun-related deaths also fell in residences might be because it was more difficult to transport around guns, which might to some degree prevent gun-related deaths in homes. In this regard, there was a small, but statistically significant effect of the restriction on gun-related deaths in females in residences, but not in public areas. This finding is important because violent deaths in women in the home are often related to domestic violence and previous research has not aimed to determine the effectiveness of gun control on intimate partner violence in middle-income countries.^38^ Research in the United States in 2006 found a similar decrease in gun-related death when domestic violence offenders were restricted from access to firearms,^39^ suggesting that such gun control measures may reduce domestic gun-related violence to a limited, but important extent. More research is needed in these regards.

Our results suggest that the effects on mortality of permanent and temporary restrictions on gun-carrying are of a similar magnitude, however more research is needed. Previous research has found that mortality rates dropped between 14% and 22% in the days when temporary restrictions were imposed.^23^^–^^25^ Our findings are also similar those in South Africa where the Firearms Control Act was reported to have reduced gun-related deaths by 13.6%.^19^

We measured outcomes (mortality rates) before and after the enactment of the restriction on carrying guns, rather than its implementation. To measure implementation, enforcement levels must be measured after the enactment. Assessing firearm seizures or arrests can give some measure of the level enforcement but, because the total number of people carrying guns is uncertain, this method cannot provide very accurate data on enforcement. We were not able to obtain data on gun seizures and arrests and therefore could not measure implementation levels of the restriction. Further research on the implementation of the gun-carrying restriction is needed to understand better the links between enactment and reduction of gun-related mortality rates.

We do not believe that the decrease we found in gun-related deaths could be confounded by any concurrent violence-prevention interventions that targeted social norms because of the sharp change in mortality rates between January and February 2012. Violence-prevention interventions do not bring about rapid changes as they gradually affect social norms around violent behaviour and weapon-carrying. The robustness checks support the assumption that there was no confounding in gun-related deaths around the time of the enactment of the gun-carrying restriction.

Our study has some limitations. First, two of the control cities enacted a gun-carrying restriction during 2016. These cities might have been considering a restriction before 2016, which could potentially confound the decision to implement the restriction or not, and that might reflect different patterns in the mortality rates in those cities compared with the other control cities. Also, between 2014 and 2016, mortality changed at the national level, possibly because of the ceasefire with FARC and the peace agreement. For these two reasons, we decided to restrict the study period to 2008–2014 to avoid capturing effects that are not strictly related to the city-wide permanent gun-carrying restriction. Second, even though our data set provides a listing of all reported deaths, it does not include some important individual-level variables, such as education level, which could be systematically different between cities. Third, we were not able to obtain data on enforcement levels over time, which limits our ability to discriminate between the role of the restriction itself and its enforcement. Our results are likely to reflect their joint effect. Finally, most of the gun-related homicides that take place in Colombia are likely associated with illegal and/or unregistered guns given that only 14% of the existing guns are registered.^40^ Carrying these guns is less likely to be affected by the restriction given that a person with such a gun is already carrying an illegal gun and might have already intended to violate the restriction or know how to avoid detection. However, even in this circumstance, overall gun-related mortality fell after enactment of the gun-carrying restriction, which suggests even bigger reductions in gun-related mortality are possible in settings where guns carried are more likely to be legal or registered and where enforcement of a gun-carrying restriction is stronger, as suggested by previous research in high-income countries.^6^^–^^11^

The public debate about gun control in Colombia and elsewhere often reveals a conflict between public perceptions about increased individual safety by carrying and owning guns and the evidence that access to firearms increases gun-related injuries at a population level. This conflict highlights the importance of increasing the body of evidence on gun-carrying restrictions, and gun control more generally. 

Focusing only on gun-carrying restrictions or gun control will always only have a limited effect on gun-related mortality. An integrated plan with a multisectoral approach is key to reducing violent injuries and deaths in a sustainable way.^2^^,^^14^^,^^41^

Our study contributes to this evidence and shows the effectiveness of gun-carrying restrictions in reducing gun-related deaths, which is not only relevant for Colombia, but also for other countries with high numbers of gun-related deaths. 
